# Pillars for successful operationalization of one health as an ecosystem approach: experience from a human-animal interface in the Maasai steppe in Tanzania

**DOI:** 10.1186/s42522-023-00087-0

**Published:** 2023-08-30

**Authors:** Paul Gwakisa, Janeth George, Calvin Sindato, Anibariki Ngonyoka, Happiness Nnko, Justine Assenga, Sharadhuli Kimera, Moses Ole Nessele

**Affiliations:** 1 College of Veterinary Medicine and Biomedical Sciences, Sokoine University of Agriculture, Box 3019, Morogoro, Tanzania; 2 National Institute for Medical Research, Tabora, Tanzania; 3 University of Dodoma, Dodoma, Tanzania; 4 Ministry of Livestock and Fisheries, Dodoma, Tanzania; 5 Food and Agriculture Organization of the United Nations (FAO), Country Office, Dodoma, United Republic of Tanzania

**Keywords:** One health, Operationalization, Systems approach, Vector-Borne Diseases, Tanzania

## Abstract

**Background:**

Solving complex public health challenges requires integrated approaches to health, such as One Health. A key element of the One Health approach is the interrelationship between human, animal and environmental health and the associated multistakeholder collaboration across many cultural, disciplinary, institutional and sectoral boundaries. Here we describe a pragmatic approach for One Health operationalisation basing on our long-term engagement with communities faced with health challenges in a human-livestock-wildlife interface in the Maasai steppe in northern Tanzania.

**Methods:**

Using a qualitative study design we performed an outcome mapping to document insights on results integration from our previous project. Data were collected through participatory community meetings, in-depth interviews and field observations. Field notes were coded and analysed using inductive thematic analysis.

**Results:**

We found that effective implementation of One Health interventions in complex ecosystems works best by understanding local conditions and their context and by working closely with the local people and relevant disciplinary players as one complex adaptive system. Community engagement, systems analysis, transdisciplinarity as well as political commitment played critical roles in successful operationalization of One Health. We have further emphasized that project ownership is as important to the local community as it is to the researchers. When used in combination, these elements (community engagement, systems analysis, transdisciplinarity) provide essential pillars for co-creation and maintaining collective action to set a common vision across disciplines, serving as inputs for a metrics-based toolbox for One Health operationalisation.

**Conclusion:**

Considering the novelty and complexity of One Health operationalisation, there is need also to develop scorecard-based guidance for assessment of One Health programs at local and national level. This paper proposes a framework for the optimization of an ecosystems-based One Health approach for prevention and control of Vector-Borne Diseases implemented at the local, sub-national or national level.

## Background

The impacts of zoonotic diseases are global, but the most vulnerable are the poor and marginalized people from developing countries who depend most directly on the ecosystems they live in for survival [1]. The emergence of such diseases is driven largely by socio-economic, environmental and ecological factors [2–4], with more than three-quarters of them originating from wildlife [5], with recent examples of Ebola [6] and SARS-CoV-2 [7, 8]. An improved understanding of the connectedness of the health of people, animals and their shared environment will help to achieve optimal global health outcomes.

Zoonotic and vector-borne diseases (VBDs) are an endemic problem in Tanzania, especially amongst pastoral communities, such as the Maasai people, who live in the Maasai steppe in northern Tanzania [9, 10]. Several zoonotic and VBDs affect the Maasai communities, and some of these, such as Rift Valley fever, rabies, brucellosis, anthrax and Human African Trypanosomiasis were listed into priority zoonotic diseases (PZDs) of greatest national concern for Tanzania [11–13]. The effects of zoonotic and VBDs in the Maasai steppe are likely to increase, due to socio-ecological factors, including climate variability/change, increased human and livestock populations, agricultural encroachment, competition for land and pastures between humans, livestock and wildlife and water scarcity, which all exacerbate the potential for increased disease burden at the human-animal interface.

Endemicity of zoonotic and VBDs in the Maasai steppe and elsewhere calls for coordinated, interdisciplinary approaches to address multisectoral health challenges. One such approach is the One Health (OH) approach, which has received global advocacy for studying the interdependence of health of humans, animals, and ecosystems [14]. To mainstream OH for global preparedness to prevent, predict, detect, and respond to global health threats and promote sustainable development, recently the Food and Agriculture Organization of the United Nations (FAO), the World Organisation for Animal Health (WOAH, formely founded as OIE), the United Nations Environment Programme (UNEP) and the World Health Organization (WHO) welcomed a newly formed operational definition of OH as an integrated, unifying approach that aims to sustainably balance and optimize the health of people, animals and ecosystems [15]. It recognizes OH as a collaborative, multisectoral, and transdisciplinary approach, working at the local, national, regional and global levels, with the goal of achieving optimal health outcomes that recognize the interconnection between people, animals, plants, and their shared environment. The approach mobilizes multiple sectors, disciplines and communities at varying levels of society to work together to foster well-being and tackle threats to health and ecosystems while addressing the collective need for clean water, energy and air, safe and nutritious food, taking action on climate change, and contributing to sustainable development [16].

Applying an OH approach to optimize zoonotic and VBDs prevention and control programs can save lives by improving efficient use of resources and the quality and timeliness of healthcare delivery [17, 18]. Despite increasing awareness of the OH approach, its practical implementation is hindered by a lack of methodological operationalisation and evaluation metrics [19–21] as well as lack of communication and coordination between human health, animal health, and environment sectors. Although awareness of OH as an approach is steadily growing, unless this is translated into action, the world remains vulnerable to future zoonotic outbreaks, considering the recent global devastating effects of the COVID-19 pandemic [22]. So far, OH implementation has been a major challenge in many countries [23–25]. The main challenges are mostly operational due to inadequate resources (finances, infrastructure and personnel).

In this communication, we describe a pragmatic approach for OH operationalization following our long-term exploration of real-life health challenges and opportunities in local community settings at a human-livestock-wildlife interface in the Maasai steppe in Tanzania. We report that implementation of OH may work best by understanding local conditions and their context and by working closely with the local people as key boundary partners in addition to involvement of multiple disciplinary players. The key findings are presented here as essential pillars for successful implementation of OH in a community.

## Methods

### Setting

**Fig. 1:**
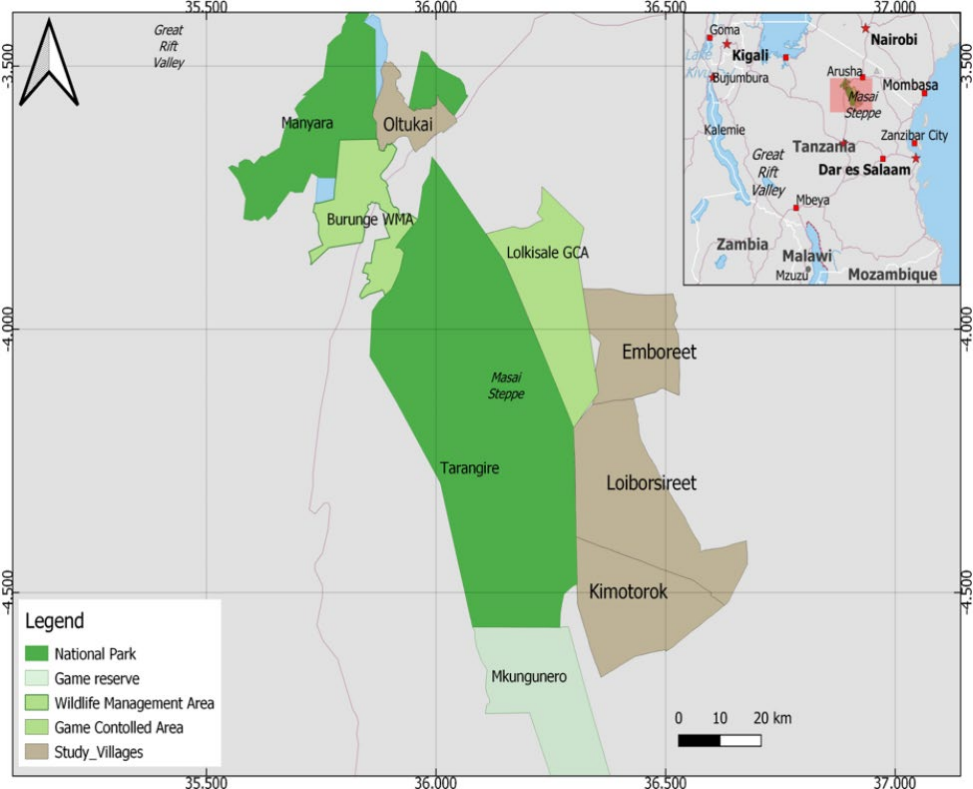
Map of the Maasai steppe in Tanzania

The Maasai steppe (3°40’ and 4°35’ South, 35°50’ and 36°20’East) includes protected areas; Tarangire National Park (TNP), Manyara National Park (MNP) and Simanjiro plains, with semi-arid vast open wooded savannah and seasonal swampy areas in northern part of Tanzania (Fig. 1). The study area was Emboret village, located in a wildlife corridor, which is dominated by Maasai communities who mainly depend on livestock as their source of livelihood and social values(22). From 2013 to 2018, we engaged in work supported by WHO/TDR/IDRC under the programme entitled ‘*Population health vulnerabilities to vector-borne diseases: increasing resilience under climate change conditions in Africa’.* We conducted a participatory multidisciplinary community-based study, targeting sustainable solutions for vector control for integration into daily practices within the community. By closely engaging, consulting, listening to local opinions and exploring best practices of the Maasai people, we obtained an understanding and appreciation of traditional means of adaptation to zoonotic diseases and climatic variability/changes, enabling local communities to enhance their resilience to VBDs, with emphasis to African Trypanosomiasis [26–31].

Two years after the project ending (2020), we visited the Maasai community in Emboret village to document how earlier study interventions for vector control impacted livelihoods. Using a qualitative study design we performed an outcome mapping to document insights on results integration from our previous project. The outcome mapping exercise enabled us to conduct a retrospective analysis for piloting operational methods for OH.

Data collected through participatory community meetings, in-depth interviews and field observations were subsequently collated and used to pilot the operationalization of OH approaches under climate variability/change conditions in the Maasai steppe. Field notes were coded and analysed using inductive thematic analysis.

## Results

### Pillar 1: operationalization of one health requires a systems thinking approach

**Fig. 2:**
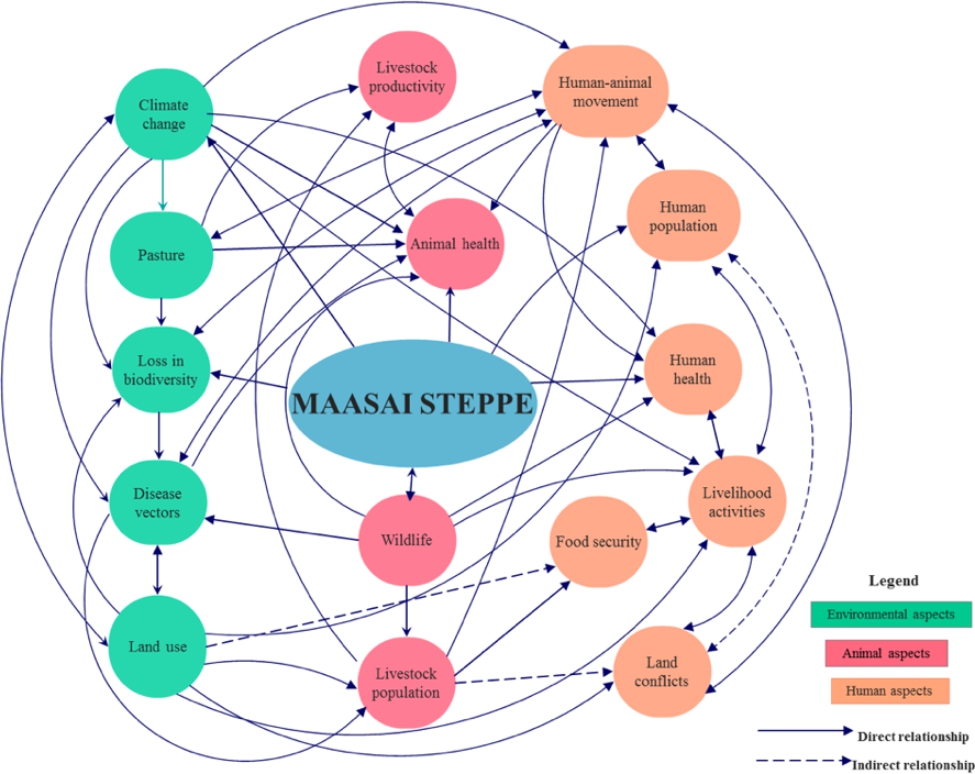
Maasai Steppe Ecosystem mapping using systems approach The figure depicts the three domains of the Maasai steppe interface. The green shapes represent environmental aspects, the pink shapes represent animal aspects and peach circles represents human aspects and livelihood within the interface. The solid and dotted arrows indicate direct and indirect influences respectively between elements

Successful operationalization of OH in any ecosystem requires a clear understanding of the socio-ecology and complexity of existing multi-faceted systems (Fig. 2). As regards the Maasai steppe, the close interconnectedness of humans, animals and their environment makes the steppe a suitable ecosystem for the inherent transmission dynamics of zoonotic diseases. Drawing from our earlier studies [10, 26–29, 31], several disease-linked factors drive the complexity of the interface ecosystem. First, prolonged droughts each year, presumed as a direct effect of climate variability/change, trigger seasonal human-animal movements (pastoralism), in pursuit of pasture and water. Pastoralism perceptibly affects livelihoods, food security as well as human and animal health and their social attributes. Second, long dry seasons and erratic rainfall in the Maasai steppe each year result in loss of biodiversity and growth of toxic invasive plants (weeds), thus limiting the quantity and quality of pasture. This in turn reduces livestock productivity, enhances livestock susceptibility to opportunistic diseases and threatens household income. Third, the long dry seasons force the Maasai herders to encroach into protected areas close to wildlife as they look for suitable land for agriculture and pasture. Encroaching into new lands for agriculture and livestock grazing leads to changes in land use patterns and increases population vulnerability to VBDs. Fourth, over the years, human and livestock populations in the rural semi-arid areas of the Maasai steppe have increased. In the foreseeable future this may likely lead to imminent human/livestock/wildlife conflicts. To help the Maasai people circumvent the effects of climate variability/change and enhance their resilience to VBDs, a systems thinking approach is likely to provide broad, innovative and optimal socio-ecological and health solutions.

To address the multiplicity of health issues in the human-animal interface ecosystem, Maasai community members and relevant stakeholders were engaged in consultative meetings to identify and prioritise intervention-researchable areas. Community members identified zoonotic VBDs as a top health issue in the area, and African Trypanosomiasis (AT) was singled out as an endemic health challenge above the other diseases in the community, affecting both humans (sleeping sickness, Human African Trypanosomiasis) and cattle (*Nagana*, Animal African Trypanosomiasis) [32, 33]. Although there have been no recent reports of sleeping sickness in the Maasai steppe, our previous studies have reported imminent risk of the disease in the area [26–29, 34, 35]. Furthermore, *nagana* is endemic in the area [36], and since cattle serve as reservoirs of human infective trypanosomes (*T. b. rhodesiense*) and tsetse flies are abundant in the area [35], it is prudent to prevent re-emergence of sleeping sickness in the human-animal interface areas, especially in the face of climate change. The systems thinking approach, with multi-sectoral community engagement allowed us to formulate a community-wide OH partnership through which it is possible to provide all-inclusive health solutions, endorsing the feasibility of OH operationalization.

### Pillar 2: transdisciplinarity is essential for the successful implementation of one health

One Health has traditionally centered on an understanding of the interdependence of health of humans, animals, and of ecosystems, often without a clear understanding of the elements of its practicability and parameterization. Recently, there has been increased interest to parameterize and operationalize this concept to effectively address public health issues. This requirement calls for implementation research using methodological metrics, which can provide a tangible understanding of what health means and offer a way to develop and implement more effective, appropriate and acceptable strategies for disease control and prevention [37]. To achieve its practicability, OH operationalization requires not only specific education in core competencies but also methodological inputs and institutional capacities, which really is the purpose of the present communication. Such inputs require transdisciplinarity for effective addressing of health challenges. Transdisciplinarity allows recognition of positions and roles in the ecosystem, knowledge integration across relevant sectors, multi-stakeholder involvement, and linkage between diverse knowledge systems to facilitate communication across disciplinary and cultural divides [38].

In order to address health issues in the complex human-livestock-wildlife interface of the Maasai steppe, actors from diverse disciplines were involved from the project inception phase to ensure multi-sectoral representation relevant to the project research problem. Their involvement from inception phase was especially crucial to ensure that commitment of different stakeholders was attained and sustained throughout the project period. Furthermore, application of multi-method analysis and working together with the local people, allowed a deep understanding of community knowledge, perception, attitudes and practices applicable to vector-borne diseases in pastoral areas. While the local Maasai people, contributed real-life experiential knowledge and perceptions on zoonoses, and local disease control mechanisms, stakeholders from sectoral ministries (health, environment, livestock) provided a bridge between researchers and communities, important for a unified implementation framework to address OH issues. The transdisciplinary approach was keystone from the initial stages of our project to develop participatory community adaptation strategies, which were uptaken as vector control interventions at the end of the project. As a result of cross-sectoral knowledge integration, it was possible to enhance sharing of some health system resources and structures at village/ward levels. This positively impacted on the uptake of our research to action and opening up of earlier siloed actions of community-level health professionals from the veterinary, human and environment sectors.

### Pillar 3: a National One Health platform is ideal to facilitate OH operationalisation

A functional national platform with multi-sectoral technical working groups is an essential prerequisite for the successful operationalisation of OH in any country. In Tanzania, the genesis of the OH platform emanated from several inward forces prevalent in the country. Firstly, several zoonotic diseases, such as brucellosis [39–44], rabies [11, 45–47], trypanosomiasis [48–52] and anthrax [13, 53–56] are endemic in the country. Such diseases can overwhelm health systems and increase vulnerabilities of communities. An earlier outbreak of Rift Valley Fever [34] in 2006–2007 caused devastating effects on human and animal health in the country. This was a major driver for Tanzania to establish a OH platform, charged with coordination and leadership roles of imminent zoonotic disease outbreak response [25]. Accordingly, initial OH actions in the country were implemented jointly by the ministry responsible for public health and social welfare and the ministry responsible for livestock. Almost during the same time (2008–2010), several OH research consortia were formed in academic and research institutions making OH a topical agenda in the country. Secondly, National efforts to build a OH platform were further inspired by a regional initiative that established the East African Integrated Disease Surveillance Network, a tripartite partnership between academia, research groups and the government [57]. Due to the relevance of multidisciplinary approaches to tackle human, animal and environmental health issues, Tanzania formally launched a national OH Agenda in 2013. This milestone was an important tool for prioritization of OH activities and strengthening engagement and collaboration between research networks and government sectoral ministries dealing with human health, animal health and the environment. In 2015, responding to the policy statement of the East African Sectoral Council of Ministers of Health, Tanzania launched a national OH Strategic Plan (2015–2020, 2021–2025). Subsequently, in 2016 a National OH Forum (NOHF), later renamed as the National OH Platform (NOHP) was formed. The platform has been administratively placed under the Prime Minister’s Office to ensure organizational clarity, government ownership, stakeholder engagement and capacity building for response to public health threats. Its placement under the Prime Minister’s office was also meant to assure that OH coordination activities receive budget allocation. The platform is led by a National OH coordinator who coordinates a team comprising experts in public health, animal health and data management and operates through three Technical Working Groups (TWGs): Surveillance, Preparedness and Response; Research and Development (R&D); and Training, Advocacy, and Communication (TAC)(25). Existence of the coordination office in Tanzania has played a pivotal role in our first steps to work with communities on advocacy and piloting the operationalisation of One Health.

### Pillar 4. Community participation is key to successful and sustainable one health initiatives

Engaging local communities at all stages of OH initiatives is important because community members, if properly engaged, can serve as essential sentinels of surveillance for timely monitoring and communicating animal, human and environmental health. This is based on the fact that disease outbreaks typically erupt at community level before spreading further and may only be recognized by health authorities at a later stage. In this study, it was evidenced that local people in Maasai communities have developed and maintained, over years, a wealth of local knowledge, attitudes and practices to recognize and manage threats of VBDs. In order to capture and blend their local knowledge with academic knowledge, it was necessary to create and maintain an active participatory environment throughout the project implementation. Forging productive partnerships with pastoral communities required to invest in time and resources, often with logistical difficulties to reach the Maasai communities, who mostly live in remote rural areas and move long distances with their animals in search for pasture and water. Engaging Maasai communities required researchers to understand the Maasai lifestyle, which is directly pegged to tribal values of livelihood and wealth, which in turn are intertwined to the health of their cattle.

Three important lessons of relevance to sustainability of researcher-community partnerships were learnt during this study. (i) Trust building between researchers and communities is an indispensable parameter to achieving good end points in OH initiatives. Forging collaborative partnership between researchers and the communities was a continuous interactive process, rather than an endpoint. Our key observation from different villages, where we engaged communities was that trust between communities and researchers was attained gradually as teams from both sides worked together while listening to community opinions and ideas. It was important that researchers understood and respected the daily and seasonal pastoralist calendars of the Maasai community, their culture and hierarchical tribal leadership. In this way, the tribal leaders together with local government played a great role in operationalizing the partnership process and sustaining interest to project activities. (ii) Community engagement yielded honest interactions and researcher-community partnership during all phases of the project. Such engagements are instrumental for project implementation and are expected to lead to successful operationalisation of future OH initiatives in vulnerable Maasai communities. (iii) Adaptation strategies and resilience to disease and environmental threats differ from one community to another. Accordingly, it was of essence that as researchers we reciprocally adapted to conditions prevailing in local communities for maximum acceptance and setting of good working dynamics.

### Pillar 5: co-creation is a collaborative innovation process for one health interventions

In order to stimulate collaboration and ownership of OH interventions, gaining a complete picture of the ecosystem in which the Maasai people live was important. Accordingly, the concept of co-creation becomes indispensable for OH innovations in communities. In the context of OH operations, co-creation implies the collaborative innovation of new solutions or interventions involving researchers, multi-sectoral health experts and community members, whereby ideas are shared and improved together from inception to implementation stages of the innovation [58, 59]. We realized that traditionally, health research in pastoral Maasai communities has been non-participatory and unsustainable, mostly due to the non-sedentary lifestyle of the Maasai people. However, our long-term engagement and close collaboration with the Maasai community enabled building of a sustainable partnership and possibility to co-create health interventions for vector control. Our proposal to co-develop adaptation strategies for tsetse and trypanosomiasis control together with the local people received acceptance and yielded a sense of openness and ownership among all stakeholders, also partly due to the significance of VBDs in the Maasai steppe. Co-creation of collaborative vector control strategies also required our understanding of social-ecological factors comprising human and environmental factors [20, 60], which influence changes in climate, land use patterns and vector and disease dynamics. In this way, it may become possible to holistically include all elements existing in the community as one ecosystem and incorporate local knowledge in the innovation process. Table 1 shows the co-creation process followed in this study, from project inception stage to outcome mapping stage, that illustrates the pilot study for OH operationalization.

**Table 1:** Co-creation experience with the Maasai community for vector control strategies

S/N	Project stage	Co-creation principle	What was done
1	Conceptualization of project idea (2012)	Social needs and Ideation	• Dialogue with local district/village authorities and communities on problem identification and project ownership
2	Final project writes up (2013)	Ideation	• Finalization of project write-up through re-alignment of ideas and interests based on stakeholders’ inputs. • Thorough literature review
3	Inception of Project activities (2014)	Pilot test and collaborative making	• Identification of stakeholders, strategic and boundary partners • Introductory meetings to share project idea at district and village levels • Interdisciplinary team building • Interface area mapping for project activities
4	Implementation of the project activities (2014–2017)	Collaborative making and implementation	• Collaborative arrangements with partner research institutions • Data collection and processing • Participatory research with local community
5	Results feedback and dissemination (2018)	Implementation and scale-up	• Results sharing with local communities and reflection on project outputs and outcomes • Co-creation of community adaptation strategies for vector control
6	Technology sharing (2018)	Implementation and scale-up	• Development of Google interface application (smart-phone based app informing pastoralists on areas to avoid tsetse flies)
7	Policy briefing at regional and national levels (2018)	Results dissemination	• Policy-briefing to sectoral ministries at national level
8	Outcome mapping (2020)	Community engagement meetings	• Assessment of results/ innovations uptake at community level and developments post project life

Critical success factors for this study were; two-way knowledge sharing between experts and community members; researchers’ respect to tribal leaders and local culture; connecting across cultures; empowering the community; valuing local Maasai knowledge; and finding the right balance between local knowledge (sacred ecology) and academic knowledge. While each of these factors played a role to different extents for planning of OH implementation, this study has demonstrated that co-creation of health strategies in communities, who live in remote areas is achievable and, indeed, necessary to improve health outcomes for vulnerable communities.

## Concluding remarks and way forward

**Fig. 3:**
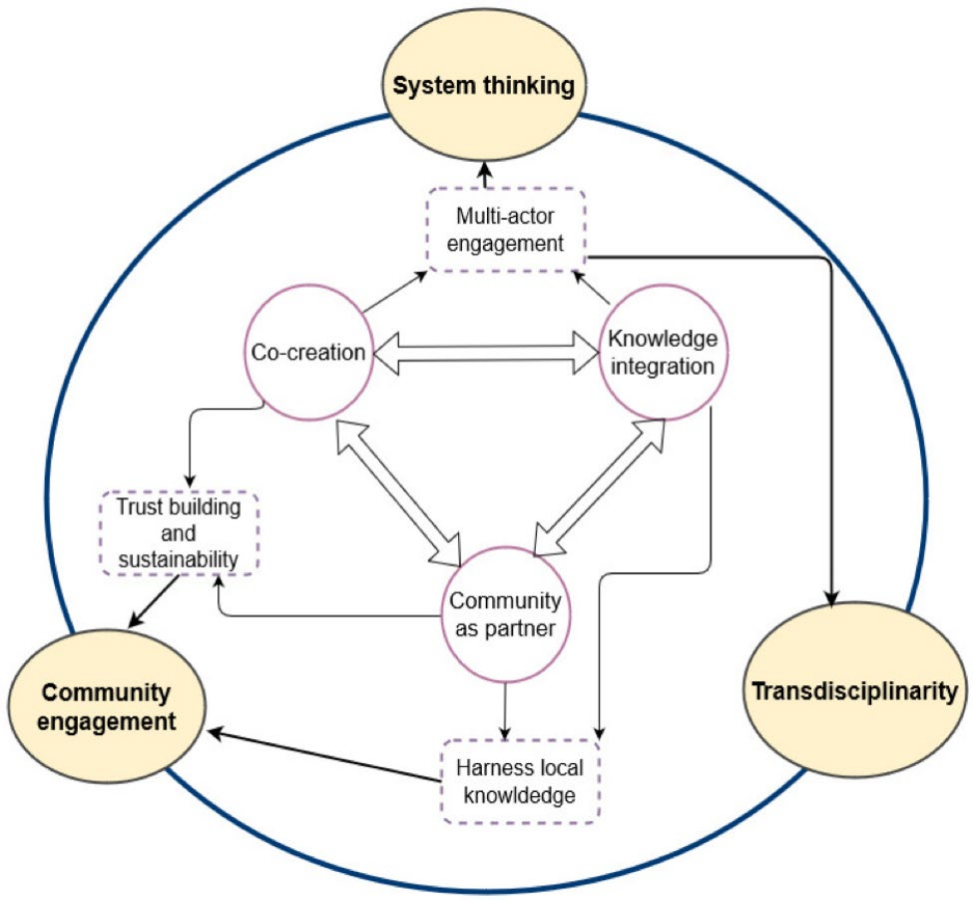
The pillars of One Health operationalisation Implementation of One Health as an ecosystem approach is anchored on three One Health pillars: Systems thinking, Transdisciplinarity and Community engagement. The three pillars are operationalized through three wheels: co-creation of interventions and their implementation processes, integration of academic and local knowledge and using communities as research partners. The wheels ensure active multi-actor engagement, trust building and sustainability of outcomes while harnessing and blending local and global knowledge

Although there have globally been many reports on the OH approach but almost none of those referred to in this paper describe operationalization of OH in real-life settings based on an ecosystem approach. This communication serves to give real-life experiences and lessons that provide guidance for development of an operational framework for OH at community level. We have illustrated the critical roles of systems analysis, transdisciplinarity, political commitment and community engagement. We have emphasized project ownership as important to the community, where work is implemented as it is to the research team and other stakeholders. When used in combination, these elements provide essential pillars for co-creation and maintaining collective action by multi-sectoral partners, building of trust and strengthening of networks for collective action towards a common vision (Fig. 3). These pillars provide methods to set a common vision across disciplines, serving as a metrics-based toolbox for systemic monitoring and feedback of OH operationalization.

Effective implementation of OH interventions will increasingly benefit from newer tools designed to allow a metrics-based assessment of OH with a consideration of environmental factors and human behaviour as one complex adaptive system. Such assessment will also serve as a management tool for building and sustaining core competencies (capacity, intervention science, risk management, impact and risk) and help to reduce impact and risks of VBDs in the face of climate change. Considering the novelty and complexity of OH operationalisation, there is need also to train diverse staff on OH operationalization as an ecosystem approach. Specifically, our current and future work is geared towards development of a scorecard-based guidance document for assessment of OH programs at local and National levels and training on fundamentals of One Health and the implementation science around it.

Lessons learnt from our work in the Maasai steppe have several implications on governance of health systems. Given the ongoing globalization, and potential for emergence of pandemics in the face of climate and socio-ecological changes, it is prudent to develop OH implementation in a broader context beyond zoonotic threats. With the tools and technologies now at hand, innovative transdisciplinary research is readily feasible to reduce health vulnerabilities of communities and ecosystems. By operationalizing OH, scientific practices for disease management with potential for translation to policy will be developed to benefit populations. If implemented correctly, OH operations should not close opportunities for subsequent operations, but rather bear an open-ended formulation to stimulate community engagement and continued interest for implementation by other multi-disciplinary stakeholders.

## Data Availability

Contact the authors for any additional information.
